# Evolutionary genetics of personality in the Trinidadian guppy II: sexual dimorphism and genotype-by-sex interactions

**DOI:** 10.1038/s41437-018-0083-0

**Published:** 2018-05-23

**Authors:** Stephen John White, Thomas Miles Houslay, Alastair James Wilson

**Affiliations:** Centre for Ecology and Conservation, University of Exeter (Penryn Campus), Cornwall, TR10 9FE UK

**Keywords:** Evolutionary genetics, Behavioural ecology

## Abstract

Sexual dimorphism in behaviour and personality has been identified in a number of species, but few studies have assessed the extent of shared genetic architecture across the sexes. Under sexually antagonistic selection, mechanisms are expected to evolve that reduce evolutionary conflict, resulting in genotype-by-sex (GxS) interactions. Here we assess the extent of sexual dimorphism in four risk-taking behaviour traits in the Trinidadian guppy, *Poecilia reticulata*, and apply a multivariate approach to test for GxS interactions. We also quantify the among-individual and genetic covariances between personality and size and growth, which are known a priori to differ between the sexes. We found significant sexual dimorphism in three of the four behaviours, although *r*_mf_ between sex-specific homologous traits was significantly <+1 for only one behaviour. Using multivariate models, we then estimated sex-specific genetic (co)variance matrices (**G**_**m**_ and **G**_**f**_) and tested for asymmetry of the cross-trait cross-sex genetic covariance structure (submatrix **B**). While **G**_**m**_ and **G**_**f**_ were not significantly different from each other overall, their respective leading eigenvectors were poorly aligned. Statistical support for asymmetry in **B** was found, but limited to a single trait pair for which the cross-sex covariances differed (i.e., COV_A(m,f)_ ≠ COV_A(f,m)_). Thus, while single- and multi-trait perspectives evidence some GxS, the overall picture is one of similarity between the sexes in their genetic (co)variance structures. Our results suggest behavioural traits related to risk-taking may lack the sex-specific genetic architecture for further dimorphism to evolve under what is hypothesised to be antagonistic selection.

## Introduction

Traits under selection should evolve in a manner dependent on the genetic variance present, the genetic covariance structure with other traits and the strength of selection (Lande 1979, Walsh and Blows 2009). While homologous traits (e.g. body size) expressed in males and females can often under sexually antagonistic (SA) selection (Reeve and Fairbairn 2001; Olsson et al. 2002; Cox and Calsbeek 2009; McPherson and Chenoweth 2012), they are likely to share a common genetic architecture (Poissant et al. 2010). Although, this shared architecture can result in conflict and thus evolutionary constraint, the prevalence of sexual dimorphism across taxa and traits suggests that sexual conflict can, at least in part, be resolved (Cox and Calsbeek 2009). Indeed, persistent SA selection is itself expected to favour mechanisms that reduce intra-locus sexual conflict, allowing the sexes to diverge towards their respective fitness optima (Lande 1980, Rhen 2000, Bonduriansky and Chenoweth 2009). These mechanisms can include sex-linkage, sex-limited trait expression, sex-specific genetic modifiers and genomic imprinting (Rhen 2000, Day and Bonduriansky 2004, Fairbairn and Roff 2006, Bonduriansky and Chenoweth 2009). However, at the whole genome level, the extent to which SA selection provides scope for further dimorphism requires characterising the magnitude of genotype-by-sex interactions (GxS). In this study we investigate sexual dimorphism and GxS interactions in a suite of risk-taking behaviours in the Trinidadian guppy, *Poecilia reticulata*.

Quantitative genetics provides several tools with which to test for and estimate GxS interactions, the presence of which implies that sex-limited genetic variance may facilitate conflict resolution and allow the divergence of the sexes (Wyman et al. 2013). The cross-sex genetic correlation (*r*_mf_) between homologous male and female traits is most commonly used to quantify the extent of sex-specific genetic variance, where

1 $$r_{{\mathrm {mf}}} = \frac{{{\mathrm {COV}}_{{\mathrm {Amf}}}}}{{\sqrt {V_{{\mathrm {Am}}}V_{{\mathrm {Af}}}} }},$$

where *V*_Am_ and *V*_Af_ are the sex-specific (additive) genetic variances and COV_Amf_ is the cross-sex genetic covariance. Typically, an *r*_mf_ of +1 is viewed as maximally constraining for sex-specific adaptation under SA selection as any increase in fitness of one sex will result in a reduction in fitness of the other sex (Bonduriansky and Chenoweth 2009, Wyman et al. 2013). Note *r*_mf_ = +1 does not imply an absolute constraint on trait evolution, as selection responses also depend on the magnitude of sex-specific additive genetic variances (*V*_Am_, *V*_Af_), which need not be equal when *r*_mf_ = +1. Only in the complete absence of GxS does it follow that both *r*_mf_ = 1 and *V*_Am_ = *V*_Af_ (Boulton et al. 2016).

Assessing GxS interactions on a trait by trait basis in this manner, while computationally and technically straightforward, give a restricted view of trait evolution. This is because natural selection acts on suites of traits simultaneously, and many of these will be genetically correlated (Lande and Arnold 1983, Walsh and Blows 2009). Multivariate approaches that account for this among-trait genetic covariance structure in the form of a **G** matrix are therefore required (Lande 1979, Blows 2007, Walsh and Blows 2009). In the context of understanding sexual dimorphism, one method has been to estimate sex-specific **G** matrices (subsequently **G**_**f**_ and **G**_**m**_) and compare them, using techniques, such as eigenvector analysis. For instance, if **G**_**f**_ and **G**_**m**_ differ in orientation and/or magnitude of their leading eigen vectors (**g**_**max**_), then continued phenotypic divergence can be possible, even if homologous traits have high pairwise *r*_mf_ (Jensen et al. 2003, Campbell et al. 2010, Wyman et al. 2013). Conversely, if the orientation of sex-specific **g**_**max**_ is similar, then this can constrain divergence between the sexes (Leinonen et al. 2011, Wyman et al. 2013).

Building on this multivariate approach, it is possible to further define a block matrix, **G**_**mf**_ that contains **G**_**m**_ and **G**_**f**_, as well as the cross-sex, cross-trait covariance submatrix usually denoted **B**. The latter can reveal avenues for constraint or divergence between the sexes not detectable in the sex-specific **G** matrices alone (Gosden et al. 2012, Wyman et al. 2013). The multivariate breeder’s equation can thus be modified to take into account SA selection (Lande 1980), such that

2 $$\left( {\begin{array}{*{20}{c}} {\Delta {\bar{{\mathbf {Z}}}}_{{{\mathbf {m}}}}} \cr {\Delta {\bar{{\mathbf {Z}}}}_{{{\mathbf {f}}}}} \end{array}} \right) = \frac{1}{2}\left[ {\begin{array}{*{20}{c}} {{{{\mathbf {G}}}}_{{{\mathbf {m}}}}} & {{{\mathbf {B}}}} \cr {{{{\mathbf {B}}}}^{{{\mathbf {T}}}}} & {{{{\mathbf {G}}}}_{{{\mathbf {f}}}}} \end{array}} \right]\left( {\begin{array}{*{20}{c}} {{{{\mathbf {\beta}} }}_{{{\mathbf {m}}}}} \cr {{{{\mathbf {\beta}} }}_{{{\mathbf {f}}}}} \end{array}} \right),$$

where $\Delta {\bar{{\mathbf {Z}}}}_{\mathbf{m}}$ and $\Delta {\bar{{\mathbf {Z}}}}_{\mathbf{f}}$ are the sex-specific vectors of predicted response for a set of traits and the **β**_**m**_ and **β**_**f**_ represent vectors of sex-specific (linear) selection gradients. The ½ coefficient accounts for both parents making equal genetic contributions to offspring of both sexes and **G**_**mf**_ is the block matrix (shown in square brackets in Eq. 2) containing submatrices **G**_**m**_, **G**_**f**_ and **B** as defined above (Lande 1980). For the simplest case of two homologous traits (x and y) expressed in both sexes, then

3 $${{{\mathbf {B}}}} = \left[ {\begin{array}{*{20}{c}} {{\mathrm {COV}}_{{\mathrm {Amf}}({\mathrm {x}})}} & {{\mathrm {COV}}_{{\mathrm {A}}({\mathrm {fx}},{\mathrm {my}})}} \cr {{\mathrm {COV}}_{{\mathrm {A}}({\mathrm {mx}},{\mathrm {fy}})}} & {{\mathrm {COV}}_{{\mathrm {Amf}}({\mathrm {y}})}} \end{array}} \right].$$

Thus, on its diagonal, **B** contains those cross-sex genetic covariances that are used to determine *r*_mf_ for each trait (here x and y), but also contains the between sex genetic covariances for each pair of non-homologous traits. Note that **B** may be asymmetric (i.e. the components above and below the diagonal in **B** are not equal, or **B** ≠ **B**^**T**^). In Eq. 3, this would be the case when the genetic covariance between male x and female y was not the same as the genetic covariance between female x and male y (i.e. COV_Amx,fy_ ≠ COV_Afx,my_). Asymmetry in **B** leads to predictions of unequal multivariate response to selection between the sexes (Steven et al. 2007, Lewis et al. 2011, Gosden et al. 2012, Berger et al. 2014).

Despite the availability of this multivariate framework, most empirical quantitative genetic studies of sexual dimorphism to date have focused on single traits (but see work on insect models by Gosden et al. 2012, Reddiex et al. 2013, Berger et al. 2014). Furthermore, GxS studies have been most commonly conducted on fitness (Chippindale et al. 2001; Brommer et al. 2007; Foerster et al. 2007), morphological (Steven et al. 2007, Leinonen et al. 2011, Potti and Canal 2011, Gosden et al. 2012) and life-history (Lewis et al. 2011) traits. Thus, while studies including average sex differences in personality traits are widespread (Aragón 2011, Gyuris et al. 2011, Koski 2011, Mainwaring et al. 2011), few also assess the presence of GxS interactions and the potential for further dimorphism to evolve (Long and Rice 2007, Berger et al. 2014). This may be due, in part, to the inherent difficulty in measuring behaviour on the large number of individuals required for quantitative genetic analysis.

Here we aim to fill this gap by assessing the extent of GxS interactions for a suite of four behaviours putatively indicative of underlying personality variation in the guppy, *P. reticulata*. We use a laboratory population of guppies, derived from a high-predation site in the Aripo River (Trinidad) and a simple open field trial (OFT) paradigm commonly used to characterise shy-bold type personality variation in fishes (Burns 2008). Here we refer to the traits collectively as ‘risk-taking behaviours’ noting that, while they should not be considered as independent, previous scrutiny of the among-individual phenotypic correlation structure does not support the idea that they all equivalent proxies of a simple shy-bold continuum (White et al. 2016). The traits included are known a priori to be significantly repeatable (White et al. 2016) and heritable in adults (White and Wilson 2018), while the genetic correlation structure has not previously been investigated (within- or between sexes).

Although, we do not estimate selection in the current study, SA selection for risk-taking behaviour is expected in this species, with the degree of conflict likely to be mediated by predation risk. Males can increase reproductive success by being highly mobile, moving between shoals to find females (Griffiths and Magurran 1998, Kelley et al. 1999, Croft et al. 2003a, b). We therefore expect male guppies to benefit from risk-taking behaviours through increased access to females. Godin and Dugatkin (1996) also found evidence that females preferred to mate with bolder males (as measured by approach distance to a predator). In contrast, risk-taking is expected to be selected against in females. When alone and away from a shoal, predation risk is high for females, with their larger size making them an energetically rewarding meal (Magurran 2005). High-shoal fidelity and tighter shoaling behaviour in females reduces predation mortality risk and increases feeding efficiency (Griffiths and Magurran 1998, Magurran and Garcia 2000, Magurran 2005, Richards et al. 2010).

The aims of this study are twofold. First, we assess the extent of sexual dimorphism for repeatable, risk-taking behaviours. We test the prediction that males will exhibit (on average) more risk-prone or ‘bold’ behaviours, before testing for dimorphism in the multivariate phenotypic (among-individual) covariance structure itself (i.e. do males and females differ in the extent or structure of (co)variation in risk-taking behaviours?). Second, we test for GxS interactions using both single trait analyses and the fully multivariate approach outlined above. While our principal focus is on risk-taking behaviours, we also expand our analyses to include size and growth traits, noting that these are known a priori to exhibit strong dimorphism in guppies, and that shy-bold type behavioural variation has been generally linked to body size across many taxa (Réale et al. 2010, Wilson et al. 2013).

## Materials and methods

### Husbandry and data collection

The data used here are derived from a larger quantitative genetics study. Most (all behavioural data, some size data) have been described elsewhere (White and Wilson 2018) along with a full description of the breeding design and pedigree structure obtained from it (see supplemental Appendix 1 of White and Wilson 2018). Thus, breeding design, general husbandry and behavioural data collection are described only briefly here.

The data set consisted of behavioural data on a total of 831 adult guppies, 616 of which were from 81 known full-sib families nested within paternal half-sibships produced between April 2013 and July 2015. To produce families, parental individuals were haphazardly sampled from a captive wild-type population (originally descended from a 2008 collection at a high-predation site in the upper Aripo river, Trinidad) at the University of Exeter, Penryn campus fish facility. After initial rearing in family groups, adult fish (average age 132 days) were tagged using visible implant elastomer (anaesthetised in buffered MS222) and put into mixed family groups of 16 (eight males, eight females). The composition of tagged groups varied according to the availability of adult fish of suitable size for tagging, but all contained representatives of at least four families. Mixing individuals from different families during development reduces the risk of common environment effects biasing additive genetic (co)variance estimates but is not possible initially as the small size of juveniles precludes safe tagging for identification.

Each adult fish underwent four open field trials (OFTs) over the course of 2 weeks. Each OFT comprised transferring a fish into an empty tank filled to 5 cm depth with water. Movement was tracked for 4 min 30 s (following a 30 s acclimation period) using Viewer software (www.biobserve.com) and a camera positioned above the tank. We chose four traits for analysis, activity (percent of the time the focal fish moved at a speed greater than the minimum threshold of 4 cm s^−1^), area covered (the total percentage of the tank explored/visited by the fish), time in middle zone (total time spent in the inner zone away from tank walls) and freezings (the total number of times movement falls below 4 cm s^−1^ for more than 2 s). A fifth trait (track length) described in White and Wilson (2018) was omitted here for purely pragmatic reasons—it was tightly correlated with activity (so carried little additional information) and reducing the number of traits facilitated multivariate model fitting (see below).

The OFT testing paradigm is widely used to assay ‘boldness’ or risk-taking behaviour in fishes with the a priori expectation that risk-prone fish will be consistently more active and exploratory, freeze less often, and be less thigmotaxic (spend less time near the edges). Order of capture within each group was recorded, as was water temperature at the end of each behavioural trial (mean of 23.7 °C). Water in the OFT tank was changed between groups. Standard length (henceforth length, measured from snout to caudal peduncle in millimeters) measures were taken at tagging, at each OFT, and 1 month after the last behavioural trial. For a subset of fish, we opportunistically collected additional size data on known age individuals at monthly intervals for up to 13 months after the last OFT. This was not possible in all cases as tanks housing groups were required for other projects in the facility. A total of 2594 behavioural trials and 4493 body size measurements were collected on 831 adults (502 females, 329 males) in a three generation pedigree structure.

### General statistical methods

Behavioural traits activity, area covered, time in middle zone and freezings were mean centred and rescaled into standard deviation units (using overall, rather than sex-specific, means and standard deviations). For time in middle zone and freezings this was done after a square-root transform to reduce positive skew and increase normality of residuals. Scaling to overall standard deviation units allows better comparison of parameters among traits and facilitates convergence of multivariate mixed models, while still preserving within-trait differences across sexes (in mean and/or variance). We denote traits by subscript m or f, when referring to male or female values specifically (e.g. Activity_m_, Activity_f_, etc.).

Data were analysed using linear mixed effect models fitted by restricted maximum likelihood in ASreml version 4 (www.vsni.co.uk). Conditional F statistics were used to test for significance of fixed effects where pertinent to biological hypotheses (e.g. to test for trait dimorphism). Note, however, that in most cases fixed effects were included principally to control for potential sources of variance not directly relevant to our hypotheses. In all behavioural models, fixed effects included temperature (of the tank water taken following each OFT), age (in days), repeat (a four-level factor to control for habituation to the OFT arena over the four-repeat trials), order caught (the order in which fish were caught from their home tank prior to the OFT, fitted as a continuous covariate) and generation (a three-level categorical effect to control for any differences in husbandry and rearing among the generations of the pedigree, see White and Wilson 2018).

Significance of random effect (co)variance components was assessed using likelihood ratio test (LRT) comparisons of nested models, with twice the difference in log-likelihoods assumed to be *χ*^2^ distributed with degrees of freedom equal to the number of parameters being tested. We caution that all *P* values presented are nominal. No corrections are made for multiple testing since, by design, statistical tests are not independent (e.g. individual traits are expected to be correlated). Random effects of group (a 40 level categorical effect to account for environmental and social sources of variation among home tanks) and fish ID were fitted to all traits in all models unless otherwise stated. To estimate genetic (co)variance parameters we used animal models (Kruuk 2004, Wilson et al.^ 2010^) further partitioning the among-fish (co)variance into additive genetic and permanent environment components. We assume an absence of maternal (identity) effects, noting that our previous study (White and Wilson 2018) showed maternal variance was non-significant for activity and bound to zero for all other OFT traits in these adult fish. Although previous analyses do suggest statistically significant effects of maternal weight and natal brood size on adult behavioural traits, their effects sizes are low (particularly relative to impacts on juvenile behaviour) and omission here has minimal impact on the sex-specific (genetic) covariance structures.

To model growth rate, we fitted random regressions of standard length over age in mixed model and animal model formulations, resulting in estimates of among-individual and additive genetic variation in both length (at average age) and growth. This reaction norm approach fits a random-by-covariate effect, allowing each level of a random effect to vary across a covariate and is an established technique in both behavioural and life-history studies (Nussey et al. 2007, Dingemanse et al. 2010, Roff and Wilson 2014). In all length/growth models, fixed effects of generation and continuous effects of age, age^2^ and age^3^ were fitted, the latter to allow a curvilinear average relationship between length and age.

### Sexual dimorphism

#### Single-trait models

To ascertain whether our traits were dimorphic on average, we fitted univariate mixed models for each behaviour and for the length/growth random regression (sexes pooled), with an additional fixed effect of sex. A significant sex effect coefficient (*P* < 0.05) was considered evidence of average trait dimorphism. We refitted the behavioural models with length as an additional covariate to determine whether average differences between the sexes in behaviour could, at least in principle, be explained entirely by size effects (given known sexual size dimorphism).

We then fitted a series of models to test for sexual dimorphism in the variance components of observed traits (as opposed to their means). For each trait (X), we fitted bivariate mixed models with X_m_ and X_f_ as responses in which we allowed variance components of interest to differ between males and females, and compared the model log-likelihood to the corresponding fit with homogeneous variance imposed. This was done first with no random effects (i.e. just residual variances), allowing test for heterogeneity of total phenotypic variance between sexes for behavioural traits and length. Note it is not possible to estimate the total phenotypic variance of growth from the random regression framework used here therefore this comparison was not done for growth. Models including fish ID and group as random effects were then fitted to test for differences in among-fish variance (Group was fitted to control for among-group variation). LRTs were used to compare the unconstrained vs. constrained (homogeneous variance across sexes) models on 1 degree of freedom (DF) for the behavioural traits and 3 DF for the length random regression.

#### Multivariate models

We next asked whether the **ID** matrix (among-individual (co)variance matrix) of OFT behaviours differs significantly between the sexes. We fitted a multivariate model with all eight sex-specific behaviours allowing estimation of **ID**_**m**_ and **ID**_**f**_ submatrices (noting that cross-sex terms are not statistically identifiable since every individual is either male or female) and compared this to a refitted model in which we imposed the condition that **ID**_**m**_ **=** **ID**_**f**_. For a more qualitative comparison, eigenvector decomposition was applied to the estimates of **ID**_m_ and **ID**_f_ matrices to see if the major axes of among-individual variation were broadly similar in males and females. More specifically, any differences in trait loadings on the first eigenvector (**id**_**max**_) were noted as well as the angle between **id**_**max**_ (the first eigen vector of **ID**) in males and females.

#### Among-individual association between personality and size

We sought to determine whether phenotypic associations between behaviour and size and/or growth differed between the sexes. Further expansion of the multivariate behavioural model to include male and female length as additional responses proved difficult, so we estimated the among-individual covariances (and corresponding correlations) with each sex-specific behaviour using a series of bivariate models. Statistical inference was by LRT comparison to constrained models in which among-individual covariance between behaviour and both size (random intercept for length) and growth (random slope) were fixed to zero.

### Quantitative genetic analyses

#### Single-trait models

Previous analysis of the OFT data with univariate animal models has shown all behaviours are significantly heritable in adults (pooled sexes, see White and Wilson 2018). Sex-specific parameters and genetic covariance structures (between traits and sexes) have not previously been estimated. For each trait we fitted bivariate animal models to estimate the genetic variance of the sex-specific sub-traits (*V*_Am_ and *V*_Af_) and genetic correlation between them (*r*_mf_). This was then compared to a model in which GxS interactions was assumed absent (*V*_Am_ = *V*_Af_, *r*_mf_ = +1). We also compared model fits to two intermediate models, one where sex-specific *V*_A_ were constrained to be equal but *r*_mf_ was free to be <+1, and a second with *r*_mf_ constrained to be +1 but sex-specific *V*_A_ free to vary. Since these intermediate models are not nested, AIC values were calculated for each model and used for additional comparison.

#### Multivariate models

Cross-sex multivariate animal models were fitted with the eight sex-specific OFT sub-traits. First we compared the sex-specific **G** matrices without estimating the cross-sex, cross-trait terms (**B**), such that we estimated **G**_**mf**_ as:

3 $${{{\mathbf {G}}}}_{{{{\mathbf {mf}}}}} = \left[ {\begin{array}{*{20}{c}} {{{{\mathbf {G}}}}_{{{\mathbf {m}}}}} & 0 \cr 0 & {{{{\mathbf {G}}}}_{{{\mathbf {f}}}}} \end{array}} \right].$$

This model was compared to one in which we impose the condition that **G**_**m**_ = **G**_**f**_ (using a LRT on 10 df). As in our comparison of **ID**_**m**_ and **ID**_**f**_, we also subjected the sex-specific submatrices to eigenvector decomposition to facilitate a qualitative comparison of trait loadings and also the angle between **g**_**max**_ of males and females. We then fitted the full multivariate model including all cross-sex cross-trait terms such that

4 $${{{\mathbf {G}}}}_{{{{\mathbf {mf}}}}} = \left[ {\begin{array}{*{20}{c}} {{{{\mathbf {G}}}}_{{{\mathbf {m}}}}} & {{{\mathbf {B}}}} \cr {{{{\mathbf {B}}}}^{{{\mathbf {T}}}}} & {{{{\mathbf{G}}}}_{{{\mathbf {f}}}}} \end{array}} \right].$$

As noted earlier, asymmetry of the upper and lower diagonals of the submatrix **B** can offer additional opportunities for sexual divergence under sex-specific selection as well as constraint. Ideally, we would have compared the log-likelihood of our full multivariate model to a constrained fit in which symmetry of **B** was imposed. We were, however, unable to obtain a stable model convergence with the latter constraint imposed. Therefore, to test for symmetry we calculated an estimate of **B**–**B**^**T**^ as a square matrix, denoted as **∆B**, noting that if **B** is symmetrical, then **B**–**B**^**T**^ = **∆B** **=** 0. In order to generate approximate 95% confidence intervals on each element of **∆B** we performed a 5000 draw parametric bootstrap on the **G**_**mf**_ matrix (following the general approach outlined in Boulton et al. 2014), implemented within the R statistical environment (R core team, 2016), estimating **∆B** for each draw. It is important to note that this matrix bootstrapping procedure assumes multivariate normality.

#### Genetic association between personality and size

As we were unable to expand the multivariate animal model further to include size/growth as well as the eight behaviours, we fitted a series of bivariate animal models between each sex-specific behaviour and length (again, modelled as a first order random regression of age for both additive and permanent environment effects). This was to determine whether behaviour-length/growth associations differed between males and females at the genetic level. As with the corresponding phenotypic analysis, the significance of genetic covariance with size/length was determined for each sex-specific behaviour using LRT and genetic covariances were standardised to correlations for easier interpretation.

## Results

### Sexual dimorphism

#### Single-trait models

**Fig. 1:**
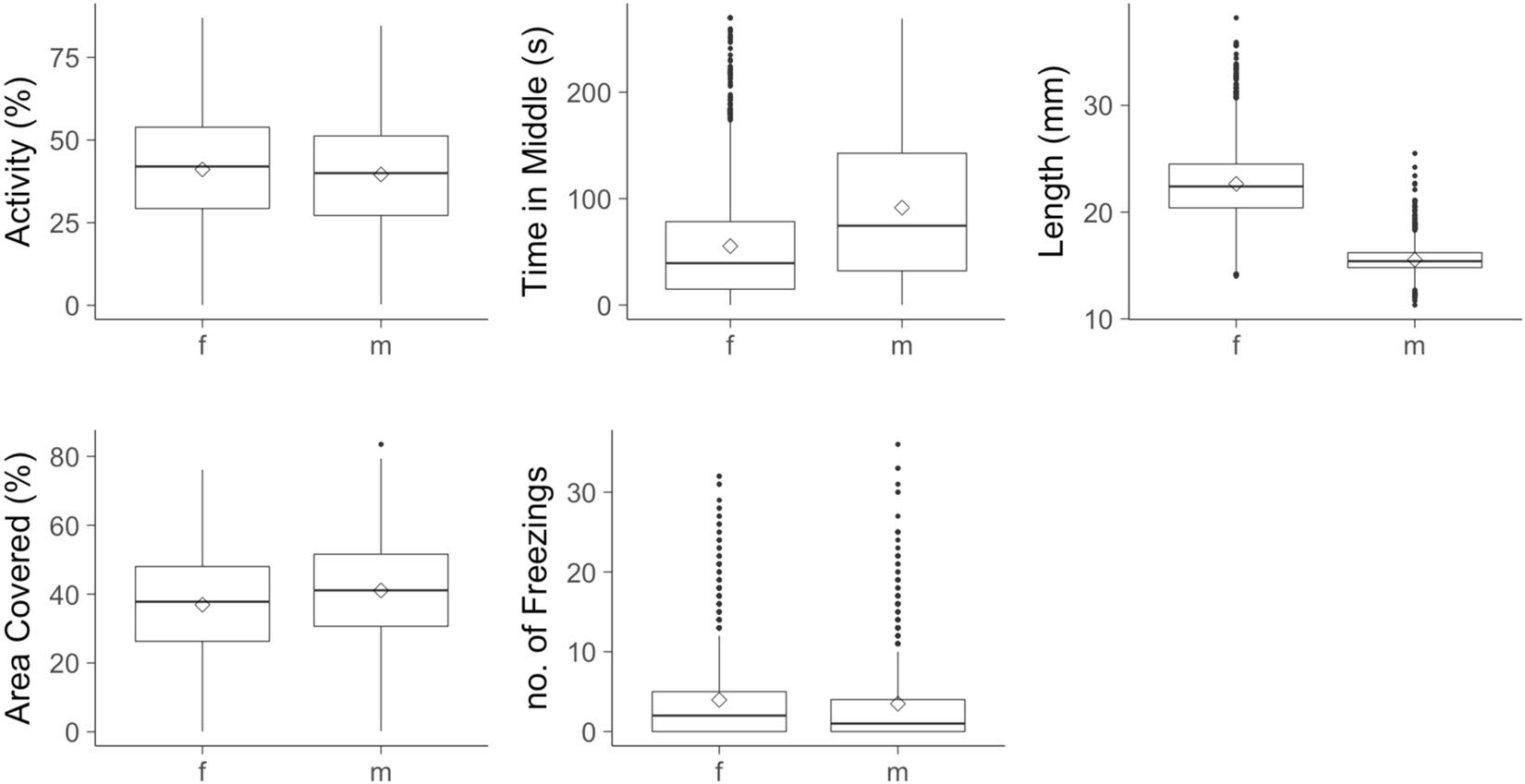
Boxplots of OFT raw data, comparing males (m) and females (f). Central horizontal line indicates the median, diamond indicates the mean

**Fig. 2:**
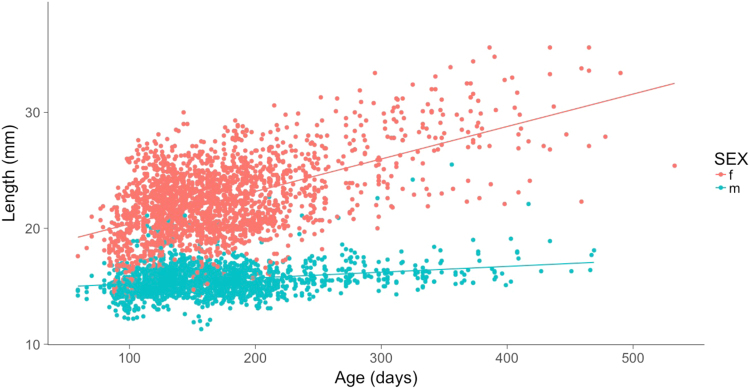
Scatterplot of individual length over age in males and females. Lines of best (linear) fit are shown for illustrative purposes only, noting that data points shown include multiple measures per individual and are non-independent

Visual inspection of raw data shows broadly overlapping distributions of male and female behavioural trait observations (Fig. 1). Nonetheless, univariate dimorphism models indicate that, conditional on other effects, all OFT traits except freezings differed significantly, on average, between the sexes. Females have significantly higher activity than males, but cover less tank area and spend less time in the middle zone (Table 1). As expected, sexual dimorphism is also present in length with females being larger on average (Fig. 1 and Table 1) and showing a steeper growth trajectory than males (Fig. 2). We note that with the addition of the covariate of length to the behavioural models, it is apparent that the dimorphism in activity could, at least in principle, be explained by size-dependence and coupled with the larger average size of females (Supplemental Table 1).

**Table 1:** Estimated effect of sex on trait means

Trait	Effect size	df	F	P
Activity	0.249 (0.053)	1779.6	21.960	<0.001
Area covered	−0.189 (0.050)	1782.3	14.38	<0.001
Time in middle	−0.507 (0.052)	1802.2	94.55	<0.001
Freezings	0.026 (0.052)	1776.6	0.24	0.621
Length	1.527 (0.035)	1745.1	1934.86	<0.001

Coefficients (with standard errors in parentheses) indicate the effect of being female relative to a male reference group. Estimates are from pooled-sex univariate animal models with (transformed) traits in standard deviation units (see main text)

Females have significantly higher activity than males, but cover less tank area and spend less time in the middle zone (Table 1)

Bivariate mixed models indicate significantly more total phenotypic variation (conditional on fixed effects) for time in middle in males (*χ*^2^_1_ = 9.68, *P* = 0.002) and for length in females (*χ*^2^_1_ = 1409.36, *P* = <0.001; Figs. 1 and 2). For the other behaviours, we found no evidence against the null hypotheses of homogeneous phenotypic variance (activity *χ*^2^_1_ = 1.04, *P* = 0.308, area covered *χ*^2^_1_ = 0.92, *P* = 0.337, freezings *χ*^2^_1_ = 0.64, *P* = 0.424; Fig. 1). Partitioning sex-specific phenotypic variance into its among- and within-individual components showed there is evidence of more among-individual variance in females than males for length/growth (*χ*^2^_3_ = 199.2, *P* = <0.001), but the sex-specific estimates of *V*_I_ are very similar for each OFT trait (Supplemental Table 2) and do not differ significantly between males and females (activity *χ*^2^_1_ = 0.254, *P* = 0.614, area covered *χ*^2^_1_ = 1.22, *P* = 0.269, time in middle *χ*^2^_1_ = 0.088, *P* = 0.767, freezings *χ*^2^_1_ = 0.16, *P* = 0.689).

#### Multivariate models

Sex-specific behavioural **ID** matrices do not differ significantly from each other (*χ*^2^_10_ = 10.62, *P* = 0.388, supplemental Table 2). The first two eigenvectors account for 64 and 26% of the behavioural variance in males and 60 and 31% in females (Table 2a). There is little difference between the sexes in how observed behaviours load onto these first two eigenvectors. For instance, in both sexes **id**_**max**_ describes an axis of among-individual behavioural variation along which activity loads antagonistically to time in middle and freezings. The angle between sex-specific estimates of **id**_**max**_ is 5.70˚, indicating very close alignment (on the scale from perfectly aligned at 0˚ to perfectly orthogonal at 90˚).

**Table 2:** Trait loadings on the first and second eigenvectors of male and female **ID** matrices (1) and **G** matrices (2)

		Male		Female	
	Trait	Eigen 1	Eigen 2	Eigen 1	Eigen 2
(1)	Activity	−0.632	0.160	−0.640	0.253
	Area covered	0.102	0.813	0.193	0.779
	Time in middle	0.575	0.388	0.537	0.408
	Freezings	0.510	−0.403	0.515	−0.404
(2)	Activity	−0.562	0.401	0.552	−0.384
	Area covered	0.320	0.644	0.584	0.377
	Time in middle	0.720	0.237	0.133	0.819
	Freezings	0.250	−0.607	−0.580	0.201

#### Among-individual association between personality and size

There is support for among-individual covariance between OFT behaviours and standard length (modelled as a random regression comprising size at average age and growth rate) although patterns are at least qualitatively different between the sexes. Area covered is the only male behaviour to significantly covary with length (Table 3, see Supplemental Table 3 for statistical inference), being negatively correlated with size at average age (weakly) and growth (moderately). In females, significant length-behaviour covariances are found for activity, time in middle and freezings. Length at average age and growth are both positively correlated with activity and negatively so with freezings (Table 3). Time in middle was weakly correlated negatively with length at average size but more strongly positively correlated with growth.

**Table 3:** Estimated sex-specific among-individual and genetic correlations between each OFT trait and length (intercept) and growth

	Trait	Male		Female	
Among-individual		Length	Growth	Length	Growth
	Activity	0.150 (0.085)	0.190 (0.130)	**0.370 (0.057)**	**0.220 (0.113)**
	Area covered	**−0.104 (0.098)**	**−0.427 (0.142)**	0.032 (0.069)	**−**0.348 (0.123)
	Time in middle	**−**0.082 (0.088)	**−**0.244 (0.130)	**−0.199 (0.066)**	**0.092 (0.124)**
	Freezings	0.031 (0.096)	**−**0.011(0.149)	**−0.205 (0.070)**	**−0.239 (0.130)**
Additive genetic	Activity	0.110 (0.370)	0.060 (0.304)	0.247 (0.216)	0.247 (0.242)
	Area covered	**−**0.205 (0.389)	**−**0.453 (0.307)	**−**0.219 (0.394)	**−**0.482 (0.293)
	Time in middle	**−**0.001 (0.387)	0.098 (0.295)	**−**0.123 (0.382)	0.167 (0.25)
	Freezings	**−**0.231 (0.375)	**−**0.049 (0.326)	**−**0.230 (0.381)	**−**0.055 (0.324)

Standard errors are in parentheses and bold font denotes parameters where covariance between behaviour and standard length is statistically significant (see Supplemental Table 3 for statistical testing)

### Quantitative genetic analyses

#### Single-trait models

Bivariate animal models of individual pairs of sex-specific homologous sub-traits provided evidence for GxS interactions for two of the five traits. The full GxS model was a significantly better fit than the constrained (no GxS) model for length/growth (*χ*^2^_7_ = 61.92, *P* = <0.001) and time in middle (*χ*^2^_2_ = 14.968, *P* = <0.001) but not the other behaviours (activity *χ*^2^_2_ = 3.912, *P* = 0.141; area covered *χ*^2^_2_ = 3.180, *P* = 0.204; freezings *χ*^2^_2_ = 0.700, *P* = 0.705). However, AIC-based comparison with intermediate models in which the constraints of homogeneous *V*_A_ and *r*_G_ = +1 were relaxed separately provided a slightly more nuanced picture (Table 4). In fact, the no GxS model was only preferred (lowest AIC) for freezings while for activity, area covered and time in middle it was the intermediate model with homogeneous *V*_A_ but *r*_mf_ <+1 allowed that was preferred (although we note in all behavioural traits ∆AIC to at least one other model was <2 such that there is little to choose between them). The fully unconstrained model (full GxS) is clearly the best fit for length/growth however, with large ∆AIC between this and all other constrained models (Table 4). Therefore, based on the combined evidence of LRTs and AIC comparisons, we conclude there was strong support for GxS interactions for length/growth and time in middle, weak support for GxS interaction in activity and area covered, and no indication of GxS interactions in freezings.

**Table 4:** Comparisons of models in which for each pair of homologous traits full GxS is allowed (unconstrained model), homogeneity of sex-specific *V*_A_ is imposed (*V*_Am_ = *V*_Af_), *r*_mf_ of +1 is imposed, or no GxS is allowed (*V*_Am_ = *V*_Af_ and *r*_mf_ = +1)

Trait	Model	AIC	∆AIC
Activity	Unconstrained	1843.26	1.85
	*V*_Am_ = *V*_Af_	1841.41	0
	*r*_mf_ = +1	1847.16	5.75
	No GxS	1843.18	1.77
Area covered	Unconstrained	2033.90	1.91
	*V*_Am_ = *V*_Af_	2031.99	0
	*r*_mf_ = +1	2036.57	4.58
	No GxS	2033.07	1.08
Time in middle	Unconstrained	1915.18	0.86
	*V*_Am_ = *V*_Af_	1914.32	0
	*r*_mf_ = +1	1926.53	12.21
	No GxS	1926.14	11.82
Freezings	Unconstrained	2311.05	3.30
	*V*_Am_ = *V*_Af_	2309.21	1.46
	*r*_mf_ = +1	2311.53	3.78
	No GxS	2307.75	0
Length	Unconstrained	−7659.74	0
	*V*_Am_ = *V*_Af_	−7652.49	7.25
	*r*_mf_ = +1	−7649.80	9.94
	No GxS	−7611.83	47.91

Shading denotes the preferred model based on AIC

#### Multivariate models

When modelled as sex-specific behaviours we found no evidence of overall significant differences between **G**_**f**_ and **G**_**m**_ (*χ*^2^_10_ = 6.78, *P* = 0.746). While reiterating the lack of significant matrix differentiation overall, visual inspection of these two submatrices of our **G**_**mf**_ estimate (Table 5) is suggestive of more additive genetic variation in male time in middle and a larger negative activity time in middle correlation. Conversely, in females there is a larger positive activity-area covered correlation. Eigenvector decomposition of **G**_**m**_ and **G**_**f**_ shows that the first (**g**_**max**_) and second eigenvectors explain 54 and 40%, and 68 and 27% of the additive genetic variation in males and females respectively (Table 2b). In males, area covered, time in middle and freezings all load positively while activity loads negatively on **g**_**max**_. In females, it is freezings that loads antagonistically with respect to activity, area covered and time in middle. In addition, the angle between male and female **g**_**max**_ is close to being orthogonal, at 80.08˚. For comparison we also calculated the angle between leading eigen vectors of the corresponding correlation matrices as 60.74˚, indicating that the lack of alignment here arises largely from differences in among-trait genetic relationships between the sexes (as opposed to differing trait-specific genetic variances since these are all set to one in the correlation matrix).

**Table 5:**
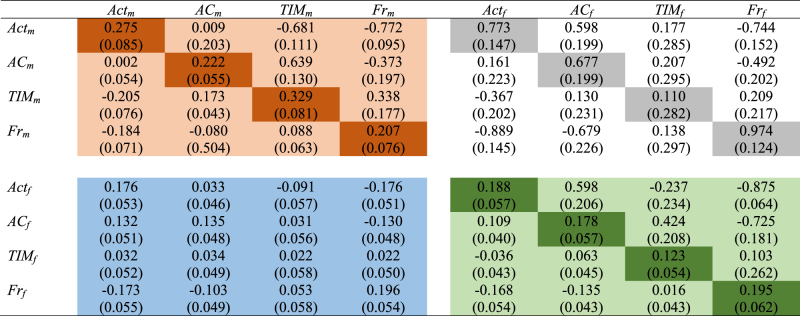
Estimated **G**_**mf**_ matrix from the full multivariate model of sex-specific OFT traits with coloured blocks corresponding to **G**_**m**_ (orange), **G**_**f**_ (green) and **B** (blue)

**G**_**m**_ and **G**_**f**_ are necessarily symmetric and shown with variances on the diagonal (dark shading), covariance below, and correlations above. **B** is not necessarily symmetric so covariances are scaled to cross-sex genetic correlations in the upper right block, with grey shading denoting the estimates of *r*_mf_ for homologous traits. Standard errors on all estimates are shown in parentheses.

The full estimate of **G**_**mf**_ also yields **B**, the cross-sex, cross-trait genetic covariance matrix. Our estimate of **B** shows that the cross-sex genetic correlations are all positive but low for time in middle (*r*_mf_ = 0.110 (0.282)), higher for activity (*r*_mf_ = 0.773 (0.147)) and area covered (*r*_mf_ = 0.677 (0.199)) and close to +1 for freezings (*r*_mf_ = 0.974 (0.124); Table 5). These effect sizes are therefore in agreement with bivariate models that evidenced GxS in time in middle and provided some (slightly equivocal) indication of *r*_mf_ < +1 in activity and area covered. Calculation of **∆B** provided some evidence for asymmetry in **B** although this is limited. Specifically, approximate 95% confidence intervals span zero for all the cross-sex elements of **∆B** except activity time in middle (95% CI = 0.005–0.245). The activity_m_—time in middle_f_ correlation being 0.177 (0.285), whereas the activity_f_—time in middle_m_ being −0.367 (0.202) (see Table 5 for the full **G**_**mf**_ matrix and Supplemental Table 4 for the **∆B** matrix).

#### Genetic associations between personality and size

Finally, bivariate animal models revealed no support for significant genetic correlations between sex-specific behaviours and length/growth in either males or females (Table 3, Supplemental Table 3).

## Discussion

Here we investigated whether personality, characterised as among-individual differences in risk-taking behaviours, is sexually dimorphic in a captive population of guppies. We also scrutinised the relationship between behaviour and length and growth—traits known to be sexually dimorphic in this species—before employing quantitative genetic analyses to assess the extent of GxS. We find statistical support for sexual dimorphism in behaviour and discuss this first before addressing the evidence for GxS provided by both the single trait and multivariate approaches used. In what follows, we put our results into the context of the wider quantitative genetic literature and also seek to highlight the benefits of taking a multivariate view of sexual dimorphism in behavioural traits.

### Sexual dimorphism in the guppy

Sexual dimorphism was present in OFT behaviours (except for freezing), as well as in length and growth. The latter result is already well known in guppies, with female fish tending to be larger, and having higher growth rates post maturity, while males preferentially invest in mating opportunities over growth (Bronikowski et al. 2002, Miller and Brooks 2005). Females also had significantly higher total and among-individual variation in length (and growth) than males, which is not unexpected given that mature fish were used and females are indeterminate growers (while males effectively stop growing after maturation). Larger females are more fecund, produce larger offspring (Reznick 1983, Bronikowski et al. 2002), and are preferred by males (Dosen and Montgomerie 2004, Herdman et al. 2004). Males, on the other hand, are selected for (relatively) fast maturation, to avoid loss of reproductive opportunities and are thought to gain little from larger size. Indeed, there is some evidence that smaller males are also more successful at sneak matings than their larger counterparts (Bisazza and Pilastro 1997). Thus, the observed size dimorphism is thought to be adaptive in the sense of reflecting divergent sex-specific optima (with larger size favoured in females).

Behavioural dimorphism is present, but effect sizes were more modest. For example, where mean length differed by ~1.5 SDU (of the pooled-sex distribution) between males and females, for the most dimorphic behaviour (freezings) the difference was only 0.5 SDU. In addition, behavioural dimorphism was only partially in line with our prediction that males would, on average, exhibit more risk-prone or ‘bold’ type behaviours than females within the novel OFT environment. We found that males tended to explore the tank more and spend more time in middle zone. This tendency fits with previous studies, for instance, Lucon-Xiccato and Dadda (2016) found that male guppies approached novel-objects and investigated more closely and quickly than females. Harris et al. (2010) and Irving and Brown (2013) both showed that male guppies emerged from the safety of a shelter more quickly than females, with a similar result found in the closely related poeciliid, *Brachyraphis episcopi* (Brown et al. 2007a). However, females were also more active than males and thus our prediction of how traits would differ between sexes was not fully upheld.

Our own previous work on female guppies (males were not tested) suggests that this could partially be explained by stress response. Although this interpretation is tentative (and perhaps subjective), high activity sometimes occurs because individuals swim rapidly and up and down one or two sides of the arena following introduction into the OFT. This is probably a general escape response found in many fish, with a fast-start swim profile consisting of rapid movement presumed to aid in predator escape (Walker et al. 2005; Marras et al. 2011). This can drive a multivariate profile in which high activity is coupled with relatively low exploration (area covered) and high thigmotaxis (i.e. less time in middle zone—White et al. 2016). We speculate that such a behavioural approach to risky/novel situations may be more common in females reflecting a stronger preference for finding shelter or a shoal (Griffiths and Magurran 1998, Magurran and Garcia 2000, Magurran 2005, Richards et al. 2010).

### Cross-sex similarity of multivariate behavioural variation

Average differences in a trait are just one way that the sexes can differ. We also estimated and compared sex-specific **ID** matrices to ask if the among-individual variance–covariance structure of OFT traits differed. A meta-analysis conducted by (Bell et al. 2009) found that, across taxa, there were significant sex differences in the repeatabilities of a wide variety of behaviours, with males being more repeatable than females. However, this pattern was actually reversed when mate choice was excluded from the analysis. Several recent studies have, however, reached varying conclusions as to which sex, if either, exhibits more within-individual consistency (Jenkins 2011, Hedrick and Kortet 2012, Debeffe et al. 2015).

While we found that males had higher among-individual variation in time in middle zone, there was no evidence that among-individual variation was greater in males for the other traits. Overall, trait repeatabilities were similar across sexes for homologous traits. Furthermore, multivariate analysis showed strong similarity of full **ID** matrix structure for OFT traits. Both males and females can therefore be differentiated along a similar continuum of behaviour, as shown by the low angle between male and female **id**_**max**_, on which activity loads antagonistically relative to the other traits. Consequently, and in contrast to results from a similar testing paradigm applied to sheepshead swordtails (Boulton et al. 2014), the structure of behavioural variation here is not really consistent with predictions under a simple shy-bold axis. Rather **id**_**max**_ of OFT traits in guppies describes a continuum of behavioural variation ranging from ‘active escape response’ at one extreme to an exploratory phenotype at the other. Average differences between the sexes (as discussed above) would therefore suggest that males inhabit the more exploratory or bold end of this axis, whereas females are closer to the escape response end of this axis.

While male and female **ID** matrices were strikingly similar here, we suggest wider estimation of these structures will be generally useful to understand among-individual (co)variation and multivariate sexual dimorphism. Certainly sexes can differ greatly in selection pressure, and in the contributions of social and abiotic factors to variation among individuals at single behavioural traits (Croft et al. 2006, Piyapong et al. 2010). To our knowledge, extension to multivariate phenotypes has rarely been attempted. In a study of wild chacma baboons (*Papio ursinus*), Carter et al. (2012) reported no difference between sex-specific principal components of (multivariate) responses to personality (boldness, novel object testing). In that case the PCA was applied to observed data (rather than an **ID** matrix) and so does not explicitly separate within- from among-individual covariance structure (Houslay and Wilson 2017). In contrast Fresneau et al. (2014) used bivariate mixed models to show that the among-individual correlation between handling aggression and nest defence was significant (and negative) in female blue tits *Cyanistes caeruleus*, but not in males.

### Evidence of size/growth-behaviour relationship

Links between risk-taking behaviours and body size (and/or growth) have been reported previously in fish (Brown and Braithwaite 2004; Brown et al. 2007b). Here our univariate models indicated that while dimorphisms in (mean) area covered and time in middle zone were largely size independent, higher activity in females could in principle be explained by sexual size dimorphism. Thus, while we have no evidence of a causal effect of body size on activity, it is possible that bigger individuals (which tend to be female) exhibit more active escape responses regardless of sex when placed in the OFT arena.

Treating standard length as response variable (rather than a ‘nuisance’ predictor of behaviour), we found some limited support for sex differences in among-individual correlations between size and behaviour. In males, individuals that cover more area in the OFT are smaller and grow less. In a previous study we also detected a negative correlation between area covered and growth in females from this population (White et al. 2016), but here it was not significant (though the estimate was, again, less than zero). The reason for this difference is not clear. The previous study was less powerful (just 32 females vs. 502 here) but also used larger and thus, given indeterminate growth, putatively older females. In the present case we did find that larger females tend to be more active, spend less time in middle zone and freeze less. In other words, larger females tended to display a more ‘escape response’ type behavioural profile in the OFT. It is difficult to speculate further on the causes of this, or other size-behaviour relationships found, beyond stating that we do not find a simple correspondence between high growth rate and risk-taking or bold behaviour as has been widely predicted, for example under the Pace of Life framework (Biro and Stamps 2008; Réale et al. 2010).

### Evidence for genotype by sex interactions

Our analysis provided strong evidence of GxS interactions for standard length (modelled as length and growth) and some support for the presence of sex-specific genetic variance in OFT behaviours. The former result suggests that length and growth have scope for further sexual divergence if SA selection is acting, and mirrors recent findings for size at maturity in another poeciliid (*Xiphophorus birchmanni*; Boulton et al. 2016). Our study does not allow us to determine the mechanism causing low *r*_mf_, though (Postma et al. 2011) found evidence of autosomal/X-linkage of body size in male guppies. While it has been suggested that the X chromosome is likely to accumulate sex-specific genetic variation (Gibson et al. 2002), other work on closely related fish have suggested that the Y chromosome could also play a role (Lampert et al. 2010; Boulton et al. 2016).

GxS interactions on OFT behaviours were detected, notably in relation to time in middle. However, across behaviours they were generally weak and less well supported statistically than GxS on size. In general the literature contains sparse estimates of GxS interactions for behavioural traits. However, in a study on selected lines of great tit (*Parus major*), van Oers et al. (2004) reported no difference in the amount of additive genetic variance between sexes for either exploration or boldness. Conversely, Han and Dingemanse (2017) found sex-specific genetic variances for exploration and aggression in the southern field cricket (*Gryllus bimaculatus*), as well as a low value of *r*_mf_ for the latter behaviour. While this suggest that importance of GxS interactions may vary across behaviour and species, it is clearly too early to generalise and more empirical studies are needed.

If contemporary selection favours further divergence of male and female behaviour, then the cross-sex genetic architecture is likely to be largely constraining in our behavioural traits. Sexual dimorphism coupled with moderate to high *r*_mf_ values has also been observed in other species (Han and Dingemanse 2017, Long and Rice 2007, Leinonen et al. 2011, Potti and Canal 2011) and it is important to note that the signature of historical GxS need not be permanent. For instance, while SA selection should favour mechanisms that allow divergence of the sexes (i.e. sources of GxS), following release from genetic constraint this same selection may erode sex-specific *V*_A,_ causing a return of high values of *r*_mf_ (Meagher 1992, Fairbairn and Roff 2006, Delph et al. 2011). Nonetheless, across OFT traits our results are consistent with the generally negative relationship between degree of dimorphism and *r*_mf_ (Bonduriansky and Rowe 2005, Poissant et al. 2010). For instance, freezings showed the least dimorphism and the highest cross-sex genetic correlation (sex difference of 0.026 SDU and *r*_mf_ of 0.974), while time in middle was the most dimorphic behaviour with the weakest correlation estimate (sex difference of −0.507 SDU and *r*_mf_ of 0.110).

From a single trait perspective, a moderate to high *r*_mf_ would lead us to conclude that the scope for further behavioural dimorphism to evolve under SA selection is limited. However, a multivariate approach can reveal either additional avenues for the sexes to diverge or additional constraints on independent evolution (Kruuk et al. 2008; Gosden et al. 2012; Wyman et al. 2013). While several studies have found differences in the structure of sex-specific **G** matrices (Jensen et al. 2003; Rolff et al. 2005; Steven et al. 2007; Lewis et al. 2011), our model comparisons provide no statistical support for significant differentiation of **G**_**m**_ from **G**_**f**_. Nonetheless, inspection of **G**_**m**_ and **G**_**f**_ reveals the largest qualitative differences between elements are associated with time in middle (both the additive variance, and additive covariances between activity and area covered), the behavioural trait for which GxS was best supported in single-trait models. Furthermore, we also estimate a large angle between male and female **g**_**max**_ vectors consistent with the two matrices differing in ‘shape.’ In fact, while **g**_**max**_ in males is similar to **id**_**max**_ in both sexes (described above), in females **g**_**max**_ trait loadings actually correspond to our a priori expectations for a shy-bold continuum (i.e. only freezing loading antagonistically to other behaviours). Reiterating the caveat that **G**_**m**_ and **G**_**f**_ are not significantly different from each other (and both estimates have high uncertainty), it is interesting that **ID** is at least a qualitatively better proxy for **G** in males than in females.

The final piece of support for multivariate GxS comes from our estimate of **B**, the submatrix of **G**_**mf**_ that describes the cross-sex genetic covariance structure. Though largely symmetrical, we found a difference in genetic association between activity_f_—time in middle_m_ (negative) and activity_m_—time in middle_f_ (weakly positive). Predictions of (multivariate) sex-specific selection responses can be drastically altered by asymmetry in **B**, though how this manifests is necessarily dependent on the relative angles of SA selection (Wyman et al. 2013). Here selection is not known so we cannot comment directly on the consequences here. Nor are there sufficient empirical studies estimating **B** where selection is known (or estimable) to generalise from the literature. However, (Lewis et al. 2011) initially found genetic constraints in the form of **G** deflecting the angle of response away from the direction of SA selection, but by including the **B** matrix these predicted responses are reversed for females and greatly reduced in males, resulting in extra constraint on sexual divergence. A similarly large effect was found for the cuticular hydrocarbons of *Drosophila serrata*, where consideration of **B** revealed significant constraints on continued sexual divergence compared to predictions from the sex-specific **G** matrices alone (Gosden et al. 2012).

## Conclusions

Despite strong interest in sexual dimorphism this is, to our knowledge, the first study to estimate **G**_**mf**_ for a set of behavioural traits. We suggest that wider uptake of multivariate analyses will give us a fuller picture of how behavioural dimorphism evolves (and why it sometimes may not). Here we show that guppies exhibit sexual dimorphism in size and growth, but also in average expression of heritable traits linked to risk-taking behaviour or shy-bold type personality variation. Although the structure of among-individual behavioural (co)variation (as measured by **ID**) is similar in males and females, single trait and multivariate analyses also provide evidence of some GxS interactions. These are detected as cross-sex genetic correlations of <1 in single trait analyses. In the multivariate analyses, the covariance structure of **G**_**m**_ and **G**_**f**_ were not significantly different from each other, although **g**_**max**_ was close to orthogonal. While there was one component of **B** that was asymmetrical, it was largely symmetrical on the whole. Lacking knowledge of (sex-specific) multivariate selection we cannot comment directly on how these genetic covariances will shape future evolution trajectories, although we broadly expect GxS to facilitate dimorphism under SA selection.

### Data archiving

The research data supporting this publication are openly available from the University of Exeter’s institutional repository at: 10.24378/exe.224.

## Electronic supplementary material

Supplemental table 1

Supplemental table 2

Supplemental table 3

Supplemental table 4

Appendix 1

Appendix 2
