# Supplementary material for: Evolutionary genetics of personality in the Trinidadian guppy II: sexual dimorphism and genotype-by-sex interactions
Source: Heredity (Edinb). 2018 May 23;122(1):15–28. doi: 10.1038/s41437-018-0083-0 (PMC6288163; doi:10.1038/s41437-018-0083-0)
Supplement: Supplementary file 1 — Supplemental table 1 [file 41437_2018_83_MOESM1_ESM.docx]

**Supplemental table 1**: Effect size of sex (male relative to female) from univariate models with the addition of length as a fixed covariate. Effect sizes are in SDU of transformed traits and standard errors in parentheses.

| Trait | Effect | Effect size | DF | F | P |
| --- | --- | --- | --- | --- | --- |
| *Activity* | sex | -0.039 (0.075) | 1, 1055.3 | 0.28 | 0.596 |
|  | length | 0.208 (0.039) | 1, 1382.2 | 28.59 | <0.001 |
| *Area covered* | sex | -0.170 (0.073) | 1, 1026.5 | 5.39 | 0.021 |
|  | length | -0.013 (0.039) | 1, 1291.6 | 0.12 | 0.724 |
| *Time in middle* | sex | -0.378 (0.075) | 1, 1068.8 | 25.41 | <0.001 |
|  | length | -0.093 (0.039) | 1, 1370.4 | 5.68 | 0.018 |
| *Freezings* | sex | 0.209 (0.076) | 1, 986.2 | 7.62 | 0.006 |
|  | length | -0.133 (0.040) | 1, 1211.9 | 11.09 | <0.001 |
