# Supplementary material for: Evolutionary genetics of personality in the Trinidadian guppy II: sexual dimorphism and genotype-by-sex interactions
Source: Heredity (Edinb). 2018 May 23;122(1):15–28. doi: 10.1038/s41437-018-0083-0 (PMC6288163; doi:10.1038/s41437-018-0083-0)
Supplement: Supplementary file 2 — Supplemental table 2 [file 41437_2018_83_MOESM2_ESM.docx]

**Supplemental table 2:** Estimated **I** matrix among OFT traits for a) males and b) females. Variances are on the diagonal (shaded), covariances on lower diagonal and correlations on upper diagonal. Standard errors in parentheses. Act= activity, AC= area covered, TIM=time in middle and Fr=freezings

| a) | Act_m_ | AC_m_ | TIM_m_ | Fr_m_ |  | b) | Act_f_ | AC_f_ | TIM_f_ | Fr_f_ |
| --- | --- | --- | --- | --- | --- | --- | --- | --- | --- | --- |
| *Act_m_* | 0.311 (0.043) | -0.058 (0.111) | -0.704 (0.050) | -0.797 (0.043) |  | *Act_f_* | 0.338 (0.034) | -0.061 (0.076) | -0.613 (0.047) | -0.791 (0.031) |
| *AC_m_* | -0.015 (0.028) | 0.207 (0.037) | 0.420 (0.092) | -0.176 (0.121) |  | *AC_f_* | -0.018 (0.023) | 0.260 (0.030) | 0.619 (0.051) | -0.128 (0.082) |
| *TIM_m_* | -0.215 (0.037) | 0.105 (0.031) | 0.300 (0.043) | 0.551 (0.080) |  | *TIM_f_* | -0.190 (0.026) | 0.169 (0.024) | 0.285 (0.030) | 0.464 (0.064) |
| *Fr_m_* | -0.222 (0.039) | -0.040 (0.029) | 0.151 (0.035) | 0.251 (0.044) |  | *Fr_f_* | -0.241 (0.030) | -0.034 (0.023) | 0.130 (0.024) | 0.275 (0.033) |
