# Supplementary material for: Evolutionary genetics of personality in the Trinidadian guppy II: sexual dimorphism and genotype-by-sex interactions
Source: Heredity (Edinb). 2018 May 23;122(1):15–28. doi: 10.1038/s41437-018-0083-0 (PMC6288163; doi:10.1038/s41437-018-0083-0)
Supplement: Supplementary file 3 — Supplemental table 3 [file 41437_2018_83_MOESM3_ESM.docx]

**Supplemental table 3:** Likelihood ratio tests for among-individual (a) and additive genetic (b) correlations between each OFT behaviour and standard length (modelled as a first order random regression on age). See methods text for details of modelling methods and Table 3 for correlation estimates. Act= activity, AC= area covered, TIM=time in middle and Fr=freezings

a) Among individual b) Additive genetic

| Behaviour | χ*^2^_2_* | P |  | Behaviour | χ*^2^_2_* | P |
| --- | --- | --- | --- | --- | --- | --- |
| *Act_m_* | 3.800 | 0.150 |  | *Act_m_* | 0.200 | 0.905 |
| *AC_m_* | 6.940 | 0.031 |  | *AC_m_* | 2.420 | 0.298 |
| *TIM_m_* | 3.340 | 0.188 |  | *TIM_m_* | 0.180 | 0.914 |
| *Fr_m_* | 3.340 | 0.188 |  | *Fr_m_* | 0.200 | 0.905 |
| *Act_f_* | 38.010 | <0.001 |  | *Act_f_* | 2.264 | 0.322 |
| *AC_f_* | 4.904 | 0.086 |  | *AC_f_* | 1.860 | 0.395 |
| *TIM_f_* | 9.114 | 0.010 |  | *TIM_f_* | 0.520 | 0.771 |
| *Fr_f_* | 9.466 | 0.009 |  | *Fr_f_* | 0.320 | 0.852 |
