# Supplementary material for: Evolutionary genetics of personality in the Trinidadian guppy II: sexual dimorphism and genotype-by-sex interactions
Source: Heredity (Edinb). 2018 May 23;122(1):15–28. doi: 10.1038/s41437-018-0083-0 (PMC6288163; doi:10.1038/s41437-018-0083-0)
Supplement: Supplementary file 4 — Supplemental table 4 [file 41437_2018_83_MOESM4_ESM.docx]

**Supplemental table 4:** Lower triangle of **∆B** matrix, calculated as **B-B^T^** (see main text for details). Lower and upper 95% confidence intervals from bootstrap in parentheses.

|  | *Activity* | *Area covered* | *Time in middle* |
| --- | --- | --- | --- |
| *Area covered* | 0.099 (-0.036,0.228) |  |  |
| *Time in middle* | 0.124 (0.005,0.245) | 0.003 (-0.116,0.12) |  |
| *Freezings* | 0.003 (-0.085,0.083) | 0.028 (-0.098,0.148) | 0.031 (-0.101,0.169) |
